# Provoked Vestibulodynia and Topical Treatment: A New Option

**DOI:** 10.3390/healthcare10050830

**Published:** 2022-04-30

**Authors:** Francesco De Seta, Guglielmo Stabile, Graziana Antoci, Gabriella Zito, Rossella E. Nappi

**Affiliations:** 1 Institute for Maternal and Child Health, IRCCS Burlo Garofolo, 34100 Trieste, Italy; fradeseta@gmail.com (F.D.S.); gabriella.zito@burlo.trieste.it (G.Z.); 2 Department of Medical, Surgical and Health Sciences, University of Trieste, 34100 Trieste, Italy; graziana.antoci@gmail.com; 3 Research Center for Reproductive Medicine, Gynecological Endocrinology and Menopause, IRCCS San Matteo Foundation, 27100 Pavia, Italy; renappi@tin.it; 4 Department of Clinical, Surgical, Diagnostic and Pediatric Sciences, School of Medicine, University of Pavia, 27100 Pavia, Italy

**Keywords:** vulvodynia, topical treatment, new therapy, vulvar vestibulitis syndrome, provoked vestibulodynia

## Abstract

Background: Provoked vestibulodynia is commonly associated with dyspareunia and affects 7% to 15% of women. This pathology has major implications on sexual function and quality of life, and several types of treatments are available for its management. However, a consensus has not been reached concerning the best treatment of vulvar pain. The aim of this study was to assess the efficacy and safety of a brand-new product, the vulvar emulgel Meclon^®^ Lenex, for the management of provoked vestibulodynia and non-infective vulvitis. Methods: This was a monocentric, prospective, randomized, double-blind and placebo-controlled study. We enrolled 40 women with provoked vestibulodynia; 20 patients received Meclon^®^ Lenex, whereas the remaining received a placebo. Each woman was assessed subjectively (through questionnaires) and objectively by evaluating vaginal and vulvar symptoms (Friedrichs criteria and Marinoff dyspareunia grade). We evaluated efficacy, safety, compliance and tolerability of the brand-new product vulvar gel Meclon^®^ Lenex in provoked vestibulodynia. Results: After administration of Meclon^®^ Lenex, we evaluated all parameters of the Friedrichs criteria (burning, dyspareunia, erythema, vulvar pain at the 5 o’clock position and 7 o’clock position), as well as the levels of Marinoff dyspareunia. The active treatment showed to be statistically significantly effective (*p* value ≤ 0.05) in reducing all symptoms of Friedrichs criteria, vulvar pain and Marinoff dyspareunia. Conclusion: This prospective study showed that Meclon^®^ Lenex vulvar emulgel revealed an excellent tolerability and compliance, demonstrating to be a safe and effective option in the treatment of provoked vestibulodynia and non-infective vulvitis.

## 1. Introduction

The vulva vestibule is a smooth surface that begins, superiorly, just below the clitoris and ends, inferiorly, at the posterior commissure of the labia minora. It contains the opening to the urethra and the vaginal opening. Its borders are formed by the edge of the labia minora. There is a demarcation between the vulva vestibule and the labia minora, called Hart’s lines. Hart’s lines identify the change from the vulva vestibule to the labia minora [1].

Provoked vestibulodynia (PVD) is a very common form of dyspareunia in both premenopausal and postmenopausal women. It is a variegated, chronic pain condition of unspecified etiology affecting 7% to 15% of women. PVD has sa ignificant impact on sexual activity and quality of life [2].

Possible causes include chronic recurrent bacterial vaginosis, recent use of chemical irritants or allergic drug reactions [3]. The most extensively studied risk factor is vulvovaginal candidiasis (VVC). As several studies state, inflammation, induced by (repeated) vulvovaginal Candida infection, could alter the equilibrium in the peripheral vulvar vestibular skin leading to local allodynia and hyperesthesia [4]. In a recent vaginal microbiome study, a history of yeast infections in women with low diversity types of vaginal community status (CST-1 and CST-2) appeared to be correlated with vulvar pain [5].

The relationship between microbiome and vulvodynia is unclear, but these CSTs are less expected to be associated with bacterial vaginosis. 

Histopathologic findings in patients affected by vulvar pain are consistent with a chronic, nonspecific inflammatory response that is occasionally associated with metaplasia of the minor vestibular glands [6].

The standard test for diagnosing PVD is the cotton-swab test, during which a cotton-swab is applied to various locations of the vulvar vestibule. However, there is much variation in the implementation of this test relating to the precise vestibular locations palpated, the order of palpation, and the force used during palpation [7].

In 1987, Friedrich [8] proposed the following diagnostic criteria for this condition: (a) severe pain upon vestibular touch or attempted vaginal entry; (b) tenderness to pressure localized within the vulvar vestibule; and (c) physical findings confined to vestibular erythema of various degrees.

Normally, women with this disorder report a “burning” pain that is right at the opening (vestibule) of the vagina. In more severe cases, the pain is present during normal daily activities, as well as during sex. Careful examination reveals redness and unusual sensitivity of the tissue at the opening of the vagina, similar to a “neuro-inflammatory” condition. This inflammatory response is promoted by nerves, which by sensing the pain, release chemicals, further favoring the inflammatory cascade [9].

Few therapies have shown to be effective. For instance, drugs for PVD include a combination of estradiol (estrogen hormone) and lidocaine (local anesthetic) compounded in a special preparation; such treatments are typically indicated for post-menopausal women. The use of topical estrogen in the vestibule has shown to improve or cure dyspareunia in more than 80% of postmenopausal women [10].

In some cases, medications acting on the interruption of the abnormal nerve sensitivity are used, for instance, tricyclic antidepressants and anti-epileptic drugs, which have already been shown to be effective for “neuropathic” (abnormal nerve function) pain [11].

Other therapeutic solutions are topical products, mainly moisturizing and lubricant ones [12,13]. In women with vulvovaginal atrophy, local estrogen therapy should be the first-line treatment. Estrogen deficiency may be a factor in the development of provoked vestibulodynia [2].

Topical medications, such as lidocaine 2% to 5%, are low-risk and may improve symptoms in the short term, ameliorating the sexual function. However, a randomized controlled trial of topical lidocaine has shown that the product does not give any benefit [14]. Compound topical therapies with gabapentin 6%, baclofen 2% and amitriptyline 2% applied several times a day were tested [15].

Cromolyn sodium cream (due to its effect on inhibiting mast cell activation) and capsaicin have been offered as topical options, although their efficacy is not demonstrated [2].

There are several non-systemic therapies that have been tested for vulvodynia management, including injection of corticosteroids, beta-interferon or botulinum toxin A in the vestibule, showing improvement in symptoms but limited efficacy [16,17]. 

Thus, the best option to treat PVD and non-infective vulvitis would be a non-invasive treatment, with limited side effects. That being so, we tested in our study a drug-free and hormone-free gel (Meclon Lenex). This gel has the potential to offer a quick relief from symptoms and to be an adjuvant in the repairing process of injured skin. 

Meclon Lenex is based on known natural active ingredients combined with topical film-forming agents designed to provide physical protection and prolonged tissue contact with the active ingredients, favoring, particularly, their humectant, hydrating and restoring actions. 

Meclon Lenex is a mixed compound made of glycyrrhetinic acid, hyaluronic acid, bisabolol, calendula officinalis flower extract, palmitamide MEA, melatonin, propolis extract and Opuntia ficus indica L. Mill. (cladodes). This brand-new product was developed by the company International Health Science (IHS) s.r.l., Lissone (MB), Italy, which also labelled and supplied our study products. 

The aim of our study was to evaluate the efficacy and safety of Meclon Lenex vulvar emulgel compared to a placebo in women in reproductive age diagnosed with PVD.

## 2. Materials and Methods

### 2.1. Study Design

This was a monocentric, prospective, randomized, double-blind and placebo-controlled study, which was conducted in Bangalore, Karnataka, between September 2021 and October 2021. 

This trial was registered prospectively with the Clinical Trials registry, India, with number CTRI/2021/07/034644. 

The research was conducted in accordance with the clinical research guidelines established by the Central Drugs Standard Control Organization (CDSCO) under the New Clinical Trial Rules and Regulations 2019, Ethical Guidelines for Biomedical Research on Human Participants 2006 of the Indian Council of Medical Research (ICMR), the principles enunciated in the Declaration of Helsinki 2013 and the ICH-harmonized tripartite guideline regarding Good Clinical Practice.

Our primary endpoint was to demonstrate that Meclon Lenex (test product) is superior to placebo and is efficacious in the resolution/reduction of vulvar pain/burning/itching/erythema in fertile women diagnosed with PVD and non-infective vulvitis.

Our secondary endpoint was to assess the safety of the product by adverse effects reporting, laboratory testing (hematology and biochemistry), physical examination and vital signs assessment.

### 2.2. Study Population 

After the establishment of inclusion and exclusion criteria (Table 1), we enrolled a total population of 40 subjects in the reproductive stage (age range: 18–45 years), diagnosed with PVD. The women entered the study after their informed written consent was obtained. In order to assure the blindness of the study, the placebo arm patients received their treatment as a gel contained in a tube, which exactly resembled the Meclon^®^ Lenex (test product) vulvar gel. 

Both study products, Meclon^®^ Lenex and the placebo, were centrally coded with randomization numbers as per the computer-generated simple randomization schedule. The enrolled patients were randomly divided in 2 study groups: Group 1 received the Meclon^®^ Lenex vulvar gel, and Group 2 received the placebo product.

The placebo product was a compound made of the same excipients and with the same pH but without Propulsave, zanthalene, hyaluronic acid, glycyrrhetinic acid, Calendula officinalis and Opuntia ficus-indica.

### 2.3. Determination of the Sample Size

A sample size of 18 patients per group achieved a power greater than 80% to reject the null hypothesis (no difference between the groups). The level of significance was set at 5% (two-sided). In conclusion, allowing for a 10% dropout, the total sample size was 40.

Demographics, vitals, medical history, treatment history and co-morbid conditions, if any, were recorded. Laboratory investigations (hematology and biochemistry) were conducted during the screening visit to rule out any underlying conditions. A urine pregnancy test was performed to rule out pregnancy. Vaginal and vulvar symptoms (Friedrichs criteria) were assessed and recorded in respective forms. Vulvar cotton swab test and Marinoff dyspareunia grade evaluation were performed and recorded in their respective forms. Concomitant medications, if any, were recorded in their respective forms.

For each recruited subject, the following steps were carried out:

- At day 0, both screening visit and baseline visit were conducted. Subjects on active compound and placebo were asked to apply the emulgel twice a day (a finger dose), once in the morning and once at bedtime, to the vulva and vestibule for 2 consecutive weeks. (It was suggested to apply it only on the painful points in cases of local provoked vulvodynia). For the following 2 weeks, they were asked to administer the gel only at bedtime for 3 times a week.
- During follow-up, 1, 2 and 4 weeks after recruitment and initial treatment, vulvar symptoms (Friedrich’s Criteria) and Marinoff dyspareunia grade were recorded.

All visits were performed, and data were collected by the two same investigators. The investigators were unaware of which products the patients had been using.

The Marinoff dyspareunia scale describes the pain limitations to practice sexual intercourse: 0, no limitations in sexual intercourse; 1, causes discomfort, but does not prevent sexual intercourse; 2, frequently prevents sexual intercourse; 3, completely prevents sexual intercourse. 

The efficacy assessment was established by evaluating the reduction of the below-mentioned conditions:

1. Burning sensation on a scale of 0–3 (0 = none, 1 = mild, 2 = moderate and 3 = severe)
2. Itching on a scale of 0–3 (0 = none, 1 = mild, 2 = moderate and 3 = severe)
3. Erythema on a scale of 0–3 (0 = none, 1 = mild, 2 = moderate and 3 = severe)
4. Swab test assessment was performed by a cotton swab examination at the 5 and 7 o’clock positions around the vaginal opening. Scoring was on a scale of 0–3 (0 = none, 1 = mild, 2 = moderate and 3 = severe). The localized nature of pain was confirmed by determining that all remaining cotton swab test points tested in the lower vagina, labia majora and labia minora were not painful.

Wilcoxon Signed rank sum test was used for continuous data for statistical analysis (*p*-value).

## 3. Results

Forty subjects were randomized and enrolled in our study. In Group 1 (active), patients enrolled were aged between 19 and 45 years, and in Group 2 (placebo), patients enrolled were aged 30–45 years. The demographics are shown in Table 2.

We compared all data obtained from Group 1 (active) and Group 2 (placebo) during the scheduled visits, i.e., from the initial screening to visits after 1, 2 and 4 weeks. 

**Figure 1:**
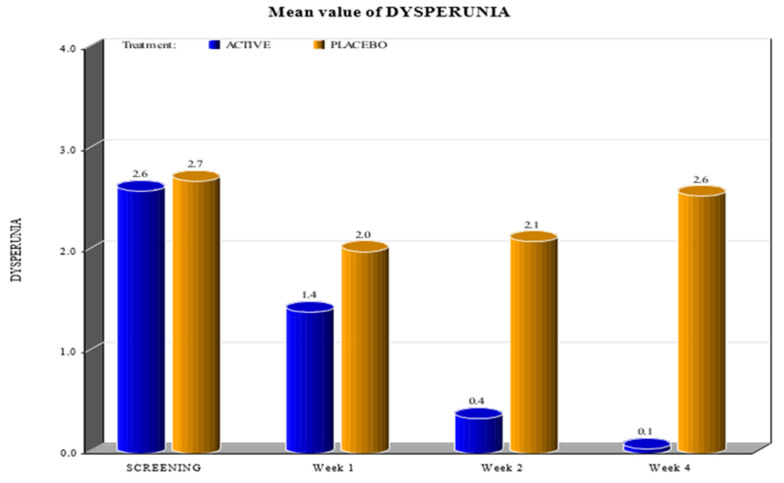
Mean value of dyspareunia (*p* value < 0.0001).

**Figure 2:**
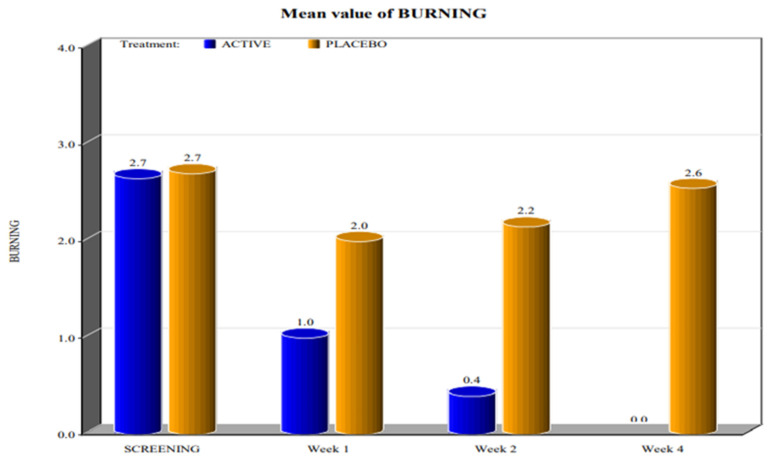
Mean value of burning (*p* value < 0.0001).

**Figure 3:**
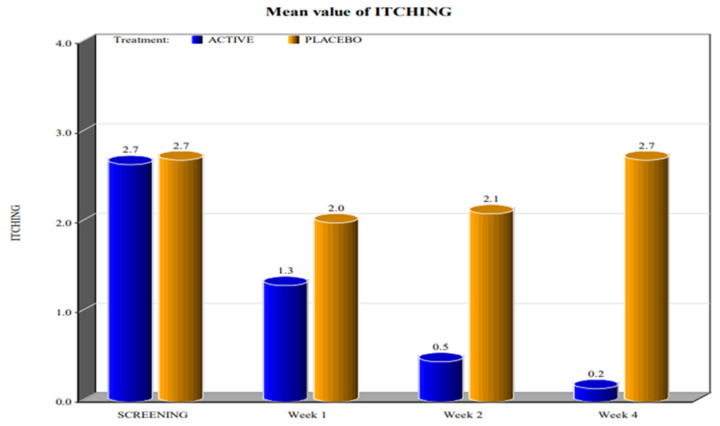
Mean value of itching (*p* value < 0.0001).

**Figure 4:**
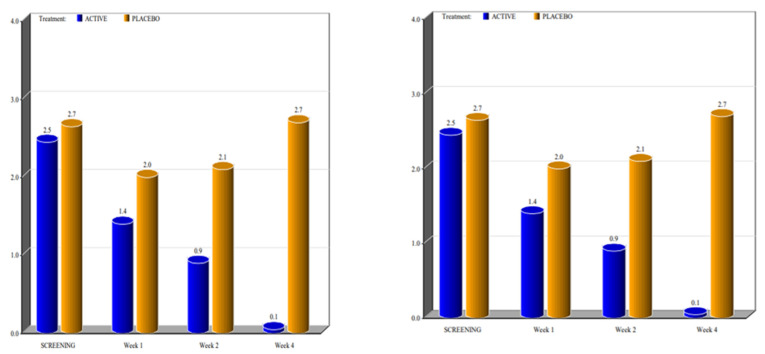
Mean value of pain determined with the swab test at the 5 o’clock (left) and 7 o’ clock (right) positions (*p* value < 0.0001).

**Figure 5:**
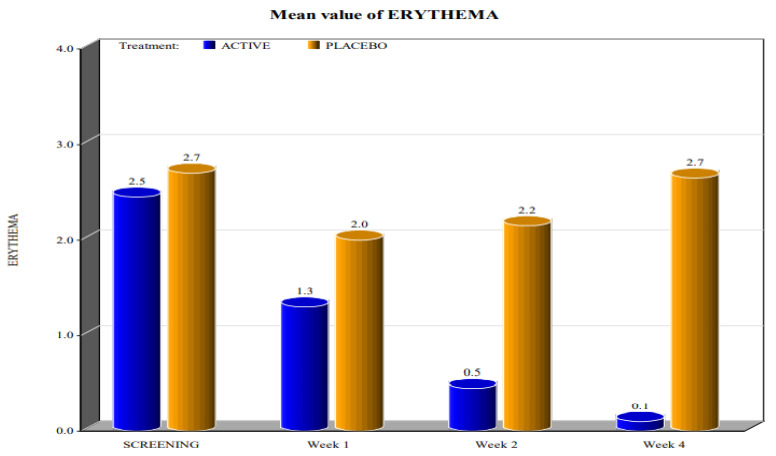
Mean value of erythema (*p* value < 0.0001).

The two groups showed significant differences in the reduction of the levels of Marinoff dyspareunia. The two groups had similar starting levels of dyspareunia but, as can be seen in Figure 1, Figure 2, Figure 3, Figure 4 and Figure 5, after only one week of products use, there were already significant differences, which further increased after 4 weeks. 

By screening patients in both active and placebo groups, we obtained the following results:

- during the screening and baseline visits, all subjects in both groups had similar levels of burning, dyspareunia, erythema and vulvar pain at the 5 o’clock and 7 o’clock positions.
- By week 1, the subjects in the active group showed a decrease in the signs of burning, dyspareunia, erythema and vulvar pain at the 5 o’clock and 7 o’clock positions when compared to placebo.
- By week 2, in subjects in the active group, the levels of burning, dyspareunia, erythema, vulvar pain at the 5 o’clock and 7 o’clock positions statistically significantly decreased with respect to the levels at screening. Instead, in subjects in the placebo group, these symptoms increased from week 1.
- By week 4, in subjects in the active group, the signs of burning, dyspareunia, erythema and vulvar pain at the 5 o’clock and 7 o’clock positions had almost disappeared in the majority of cases.

In the placebo arm, we noted a slight improvement in the first week of product use (probably due to the placebo effect), but these beneficial effects of the placebo disappeared after 4 weeks. These data confirmed that the active product significantly reduced itching, burning (with the complete disappearance of the symptom after 4 weeks), dyspareunia, erythema, and vulvar pain at the 5 o’clock and 7 o’clock positions when compared to the placebo.

For all parameters of the Friedrichs criteria—burning, dyspareunia, erythema, vulvar pain at the 5 o’clock and 7 o’clock positions—the active group showed statistically significant results when compared to the placebo group at weeks 1, 2 and 4. For all parameters in the Friedrichs criteria in both active and placebo group, the *p*-value from screening to weeks 1, 2 and 4 was highly significant (*p* value ≤ 0001).

**Figure 6:**
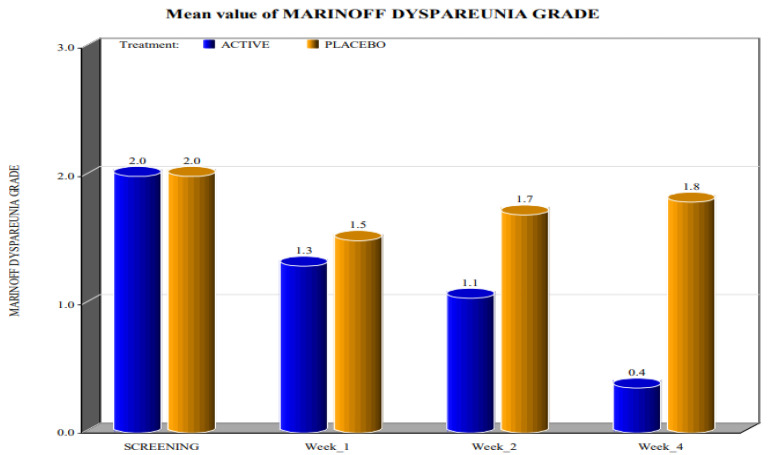
Mean values of the Marinoff dyspareunia grade. (*p* value < 0.0001).

Concerning the levels of Marinoff dyspareunia, after the use of the active product, improvements were evident (Figure 6). At the screening time, both groups had dyspareunia of grade 2. By week 1, the subjects in the active group, as well as the subjects in the placebo arm, graded their dyspareunia as 1. The same result was recorded during week 2. However, during week 4, almost all subjects in the active group graded their dyspareunia as 0, whereas for subjects in the placebo arm, the levels of Marinoff dyspareunia returned to be similar to those at the screening visit (Table 3).

These data demonstrated the high efficacy of the active product in reducing Friedrichs criteria when compared to the placebo.

Probably, this result was obtained thanks to the synergistic action of the components of Meclon Lenex:

- Hydration and protection of the vaginal tissue by reducing unpleasant symptoms such as itching, burning and irritation (zanthalene);
- Promotion of the healing process of any micro-lesions caused by tissue dryness (hyaluronic acid);
- Soothing of the skin and reduction of the inflammatory state (glycyrrhetinic acid; Calendula officinalis, Opuntia ficus-indica).

All subjects in the active as well as the placebo groups stated the excellent tolerability of the medications. All subjects were compliant with the products administration from screening visit to week 4 (Table S1—Supplementary). The safety assessed by physical examination is reported in Table S2—Supplementary. None of the subjects in the active or placebo groups had any abnormal physical examination during the whole course of the study. In the present study, none of the subjects showed any adverse event. 

## 4. Discussion

This prospective study suggests that vulvar pain in women during reproductive age progressively decreased in intensity and presence, following a treatment with the active product Meclon Lenex. 

PVD is a significant source of genital and sexual pain affecting several dimensions of women’s daily living. Indeed, PVD is often associated with comorbid physical and psychological conditions, including depressive and anxiety disorders, and rarely with more serious complications [18].

The psychological burden in women with PVD includes a lower level of happiness and poor quality of life compared with women without this painful condition [19]. 

Women with PVD suffer from experiencing high levels of sexual dysfunction and associated distress, including difficulties with desire, arousal, orgasm and satisfaction [20].

The psychological component of pain is a very important factor in the treatment of these patients and can affect the results of a therapy. In the placebo arm, we noted a slight improvement in the first week of placebo administration (probably due to the placebo effect), but these beneficial effects of the placebo disappeared after 4 weeks.

To reduce the possibility of bias, we excluded subjects with neuropathology, diagnosed psychological disorders or other comorbidities that could induce chronic pain from the trial.

Historically, different therapeutic approaches, including psychological, medical and surgical interventions, have been tried in PVD management. However, well-designed, statistically significant studies are limited. Among the therapies used over the years for vulvar pain treatment, we may list the following: a low oxalate diet, tricyclic antidepressants (TCAs), selective serotonin reuptake inhibitors (SSRIs), serotonin and norepinephrine reuptake inhibitors (SNRIs), psychosexual therapy, systemic and topic hormones and vestibulectomy. In a 2005 systematic review, most treatments for vulvar pain were described as having insufficient evidence of efficacy [21,22].

In 2016, Goldstein and colleagues [23] concluded that there was insufficient evidence to support the use of topical lidocaine, corticosteroids, or capsaicin for the treatment of localized vestibular pain. Furthermore, they underlined that evidence did not support the use of botulinum toxin A, interferon, hormonal treatments, anti-depressants, or anti-convulsants. 

Considering the insufficient evidence, the ineffectiveness of different types of drugs and the possibility that these drugs can lead to adverse effects, especially if orally administered, vaginal administration is a valid alternative route of drug delivery. It provides targeted therapy with decreased systemic adverse effects, while avoiding complications associated with oral dosing, such as interpatient variability and first-pass metabolism [24,25].

However, there is no consensus regarding the best treatment for vulvodynia. 

In our study, the active product Meclon^®^ Lenex demonstrated to be effective as an adjuvant topic therapy; it is drug- and hormone-free, with natural active ingredients combined with topical film-forming agents. Meclon^®^ Lenex showed to be statistically significantly effective (*p* value ≤ 0.05) in reducing all symptoms of Friedrichs criteria and Marinoff dyspareunia and displayed a good tolerability and safety. 

The major limitation of our study is represented by the small sample size. Another limitation could be the difference in median age between the two groups (29 in group 1, 38 in the group 2). Patients’ hormone levels, which vary over the years, can lead to alterations of the vaginal flora that induce chronic vulvo-vaginal inflammation and local allodynia and hyperesthesia. 

However, the strength of our study is based on the presence of two very homogeneous groups, from the point of view of symptoms at baseline level and clinical history. Furthermore, it was a randomized, double-blind and placebo-controlled study. The medical examinations were performed at the same time for the two groups and by the same two investigators. The pain scales for the Marinoff criteria were well defined for investigators and patients.

However, further and more extensive data are needed in order to confirm our results. 

## 5. Conclusions

Meclon^®^ Lenex is a cosmetic emulgel developed by IHS s.r.l., with a local action and without pharmacological effects. The results suggest that Meclon^®^ Lenex may be effective in promoting symptom remission of vulvitis, such as itching and burning; it promotes the natural healing of vulvar skin and vestibular mucosa by enhancing both the hydration and an adequate lubrication of these tissues.

## Figures and Tables

**Table 1:** Inclusion and exclusion criteria.

**Inclusion Criteria:**	**Exclusion Criteria were:**
**1**. Patient able to give informed consent; **2**. Age between 18 and 45 years; **3**. Patients diagnosed with PVD and/or non-infective vulvitis who had at least one of the following: a history of vulvar pain/vulvar vestibule erythema/vulvar tenderness upon the cotton swab test (“Friedrich’s Criteria”)/presence of symptoms for ≥3 months; **4**. Patients with coexisting vulvovaginal candidiasis, on maintenance with azole and negative follow-up fungal cultures, yet still fulfilled the criteria for vulvar vestibulitis.; **5**. Patients not taking any other therapy for PVD for at least a month before enrolment; **6**. Understanding and acceptance of the obligation to return to all scheduled visits and follow-ups;	**1**. Pregnant and lactating women; **2**. Subjects with a clinically significant history of co-morbid conditions (diabetes, immunodeficiency, HIV, HPV), neuropathology, psychological disorders, other comorbidities that could induce chronic pain, atrophic vaginitis, dermatitis such as vulvar dystrophy, eczema or pathogens such as culture-/smear-proven *Candida* spp. or Herpes simplex; **3**. Subjects on hormone replacement therapy or chemotherapy or radiotherapy; **4**. Subjects participating in any other clinical studies or who participated in any clinical trial 3 months prior this trial; **5**. Genital bleeding of unknown cause; **6**. Known history of malignancy; **7**. Known history of drug or alcohol abuse; **8**. Psychiatric disorders precluding informed consent; **9**. Any other medical condition that the investigators felt would compromise the study; **10**. Known hypersensitivity to, or intolerance of, the study products or their formulation excipients;

**Table 2:** Legend: BMI (body mass index), Min (minimum), Max (maximum).

Parameter/Statistics	Group 1 (Active)	Group 2 (Placebo)
**Age (Years)**		
n	20	20
Mean (SD)	31.1 (8.96)	38.4 (5.22)
Median	29.0	38.0
Min, Max	19, 45	30, 45
**Height (cm)**		
n	20	20
Mean (SD)	163.3 (4.57)	165.5 (5.84)
Median	162.0	165.0
Min, Max	157, 172	157, 175
**Weight (kg)**		
n	20	20
Mean (SD)	70.4 (5.45)	70.3 (7.34)
Median	70.0	68.5
Min, Max	61, 84	58, 81
**BMI (kg/m^2^)**		
n	20	20
Mean (SD)	26.427 (2.3543)	25.693 (2.8493)
Median	27.195	26.220
Min, Max	22.65, 30.84	21.16, 31.64
**Past Medical History, n [%]**		
Nil	20 (100.0)	20 (100.0)
Type 2 Diabetes	0 (0.0)	0 (0.0)
**Treatment History, n [%]**		
Nil	20 (100.0)	20 (100.0)
**Co-morbid Conditions, n [%]**		
Nil	20 (100.0)	20 (100.0)
**Concomitant Medications, n [%]**		
Nil	20 (100.0)	20 (100.0)

**Table 3:** Reduction of Marinoff dyspareunia from screening to week 1, week 2 and week 4.

Parameter/Statistics	Visit	Group 1 (Active)	Group 2 (Placebo)
MARINOFF DYSPAREUNIA			
n	Screening	20	20
Mean (SD)	Screening	2.0 (0.00)	2.0 (0.00)
Median	Screening	2.0	2.0
Min, Max	Screening	2, 2	2, 2
n	Week 1	20	20
Mean (SD)	Week 1	1.3 (0.47)	1.5 (0.51)
Median	Week 1	1.0	1.5
Min, Max	Week 1	1, 2	1, 2
n	Week 2	20	20
Mean (SD)	Week 2	1.1 (0.22)	1.7 (0.47)
Median	Week 2	1.0	2.0
Min, Max	Week 2	1, 2	1, 2
n	Week 4	20	20
Mean (SD)	Week 4	0.4 (0.49)	1.8 (0.41)
Median	Week 4	0.0	2.0
Min, Max	Week 4	0, 1	1, 2

## Data Availability

The original contributions presented in the study are included in the article; further inquiries can be directed to the corresponding author.

## Supplementary Materials

The following supporting information can be downloaded at: https://www.mdpi.com/article/10.3390/healthcare10050830/s1, Table S1: Drug compliance; Table S2: Descriptive statistics for physical examination.
