# Supplementary material for: Provoked Vestibulodynia and Topical Treatment: A New Option
Source: Healthcare (Basel). 2022 Apr 30;10(5):830. doi: 10.3390/healthcare10050830 (PMC9142053; doi:10.3390/healthcare10050830)
Supplement: Supplementary file 1 [file healthcare-10-00830-s001.zip › healthcare-1696015-supplementary.pdf]

healthcare

Supplementary Materials

Table S1. Drug compliance.

Parameter/Statistics	Active	Placebo
Overall IP Compliance %		
n	20	20
Mean (SD)	100.0(0.00)	100.0(0.00)
Median	100.0	100.0
Min, Max	100,100	100,100

Table S2. Descriptive statistics for physical examination.

Parameter/Statistics	Visit	Active	Placebo
General Appearance			
Normal	Screening	20(100.0)	20(100.0)
Abnormal	Screening	0(0.0)	0(0.0)
Not Done	Screening	0(0.0)	0(0.0)
Normal	Visit 3	20(100.0)	20(100.0)
Abnormal	Visit 3	0(0.0)	0(0.0)
Not Done	Visit 3	0(0.0)	0(0.0)
Normal	Visit 4	20(100.0)	20(100.0)
Abnormal	Visit 4	0(0.0)	0(0.0)
Not Done	Visit 4	0(0.0)	0(0.0)
Normal	Visit 4	20(100.0)	20(100.0)
Abnormal	Visit 4	0(0.0)	0(0.0)
Not Done	Visit 4	0(0.0)	0(0.0)
**Head, Eyes, Ears, Nose, and Throat**			
Normal	Screening	20(100.0)	20(100.0)
Abnormal	Screening	0(0.0)	0(0.0)
Not Done	Screening	0(0.0)	0(0.0)
Normal	Visit 3	20(100.0)	20(100.0)
Abnormal	Visit 3	0(0.0)	0(0.0)
Not Done	Visit 3	0(0.0)	0(0.0)
Normal	Visit 4	20(100.0)	20(100.0)
Abnormal	Visit 4	0(0.0)	0(0.0)
Not Done	Visit 4	0(0.0)	0(0.0)
Normal	Visit 4	20(100.0)	20(100.0)
Abnormal	Visit 4	0(0.0)	0(0.0)
Not Done	Visit 4	0(0.0)	0(0.0)
**Abdomen**			
Normal	Screening	20(100.0)	20(100.0)
Abnormal	Screening	0(0.0)	0(0.0)
Not Done	Screening	0(0.0)	0(0.0)
Normal	Visit 3	20(100.0)	20(100.0)
Abnormal	Visit 3	0(0.0)	0(0.0)
Not Done	Visit 3	0(0.0)	0(0.0)
Normal	Visit 4	20(100.0)	20(100.0)
Abnormal	Visit 4	0(0.0)	0(0.0)
Abnormal	Screening	0(0.0)	0(0.0)
Not Done	Screening	0(0.0)	0(0.0)
Normal	Visit 3	20(100.0)	20(100.0)
Abnormal	Visit 3	0(0.0)	0(0.0)
Not Done	Visit 3	0(0.0)	0(0.0)
Normal	Visit 4	20(100.0)	20(100.0)
Abnormal	Visit 4	0(0.0)	0(0.0)
Not Done	Visit 4	0(0.0)	0(0.0)
Normal	Visit 4	20(100.0)	20(100.0)
Abnormal	Visit 4	0(0.0)	0(0.0)
Not Done	Visit 4	0(0.0)	0(0.0)
**Gastrointestinal**			
Normal	Screening	20(100.0)	20(100.0)
Abnormal	Screening	0(0.0)	0(0.0)
Not Done	Screening	0(0.0)	0(0.0)
Normal	Visit 3	20(100.0)	20(100.0)
Abnormal	Visit 3	0(0.0)	0(0.0)
Not Done	Visit 3	0(0.0)	0(0.0)
Normal	Visit 4	20(100.0)	20(100.0)

Abnormal	Visit 4	0(0.0)	0(0.0)
Not Done	Visit 4	0(0.0)	0(0.0)
Normal	Visit 4	20(100.0)	20(100.0)
Abnormal	Visit 4	0(0.0)	0(0.0)
Not Done	Visit 4	0(0.0)	0(0.0)
	**Musculoskeletal**		
Normal	Screening	20(100.0)	20(100.0)
Abnormal	Screening	0(0.0)	0(0.0)
Not Done	Screening	0(0.0)	0(0.0)
Normal	Visit 3	20(100.0)	20(100.0)
Abnormal	Visit 3	0(0.0)	0(0.0)
Not Done	Visit 3	0(0.0)	0(0.0)
Normal	Visit 4	20(100.0)	20(100.0)
Abnormal	Visit 4	0(0.0)	0(0.0)
Not Done	Visit 4	0(0.0)	0(0.0)
Normal	Visit 4	20(100.0)	20(100.0)
Abnormal	Visit 4	0(0.0)	0(0.0)
Not Done	Visit 4	0(0.0)	0(0.0)
	**Neurological**		
Normal	Screening	20(100.0)	20(100.0)
Abnormal	Screening	0(0.0)	0(0.0)
Not Done	Screening	0(0.0)	0(0.0)
Normal	Visit 3	20(100.0)	20(100.0)
Abnormal	Visit 3	0(0.0)	0(0.0)
Not Done	Visit 3	0(0.0)	0(0.0)
Normal	Visit 4	20(100.0)	20(100.0)
Abnormal	Visit 4	0(0.0)	0(0.0)
Not Done	Visit 4	0(0.0)	0(0.0)
Normal	Visit 4	20(100.0)	20(100.0)
Abnormal	Visit 4	0(0.0)	0(0.0)
Not Done	Visit 4	0(0.0)	0(0.0)
